# 3-{(*E*)-[(4-Formyl­phen­yl)iminium­yl]meth­yl}naphthalen-2-olate

**DOI:** 10.1107/S1600536810051822

**Published:** 2010-12-15

**Authors:** Abeer Mohamed Farag, Siang Guan Teoh, Hasnah Osman, Madhukar Hemamalini, Hoong-Kun Fun

**Affiliations:** a School of Chemical Sciences, Universiti Sains Malaysia, 11800 USM, Penang, Malaysia; b X-ray Crystallography Unit, School of Physics, Universiti Sains Malaysia, 11800 USM, Penang, Malaysia

## Abstract

The title Schiff base compound, C_18_H_13_NO_2_, is a zwitterion, with the naphthol hy­droxy group deprotonated and the imine N atom protonated. It adopts an *E* configuration about the central C=N double bond. The dihedral angle between the naphthyl ring system and the benzene ring is 1.73 (11)°. An intra­molecular N—H⋯O hydrogen bond generates an *S*(6) ring motif. In the crystal, adjacent mol­ecules are connected by inter­molecular C—H⋯O hydrogen bonds, forming a supra­molecular ribbon along the *b* axis.

## Related literature

For details and applications of condensation reactions, see: Alsalim *et al.* (2010 ▶); Wadher *et al.* (2009 ▶); Abou-Melha & Faruk (2008 ▶); Sondhi *et al.* (2006 ▶). For hydrogen-bond motifs, see: Bernstein *et al.* (1995 ▶).
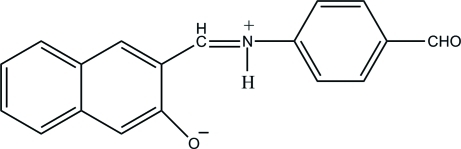


## Experimental

### 

#### Crystal data

C_18_H_13_NO_2_
*M* *_r_* = 275.29
Monoclinic, 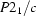

*a* = 7.3685 (8) Å
*b* = 12.7437 (13) Å
*c* = 14.4586 (15) Å
β = 91.979 (7)°
*V* = 1356.9 (2) Å^3^
*Z* = 4
Mo *K*α radiation
μ = 0.09 mm^−1^
*T* = 296 K
0.86 × 0.08 × 0.07 mm

#### Data collection

Bruker SMART APEXII CCD area-detector diffractometer
Absorption correction: multi-scan (*SADABS*; Bruker, 2009 ▶) *T*_min_ = 0.928, *T*_max_ = 0.994
11574 measured reflections
2394 independent reflections
1416 reflections with *I* > 2σ(*I*)
*R*_int_ = 0.054

#### Refinement

*R*[*F* ^2^ > 2σ(*F* ^2^)] = 0.064
*wR*(*F* ^2^) = 0.171
*S* = 1.04
2394 reflections
194 parameters
H atoms treated by a mixture of independent and constrained refinement
Δρ_max_ = 0.13 e Å^−3^
Δρ_min_ = −0.23 e Å^−3^

### 

Data collection: *APEX2* (Bruker, 2009 ▶); cell refinement: *SAINT* (Bruker, 2009 ▶); data reduction: *SAINT*; program(s) used to solve structure: *SHELXTL* (Sheldrick, 2008 ▶); program(s) used to refine structure: *SHELXTL*; molecular graphics: *SHELXTL*; software used to prepare material for publication: *SHELXTL* and *PLATON* (Spek, 2009 ▶).

## Supplementary Material

Crystal structure: contains datablocks global, I. DOI: 10.1107/S1600536810051822/is2641sup1.cif

Structure factors: contains datablocks I. DOI: 10.1107/S1600536810051822/is2641Isup2.hkl

Additional supplementary materials:  crystallographic information; 3D view; checkCIF report

## Figures and Tables

**Table 1:** Hydrogen-bond geometry (Å, °)

*D*—H⋯*A*	*D*—H	H⋯*A*	*D*⋯*A*	*D*—H⋯*A*
N1—H1*N*1⋯O1	1.00 (3)	1.68 (3)	2.551 (3)	143 (2)
C8—H7⋯O2^i^	0.93	2.54	3.399 (4)	153
C17—H17*A*⋯O2^i^	0.93	2.57	3.453 (4)	159

Symmetry code: (i) 
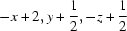
.

**Fig. 1.**
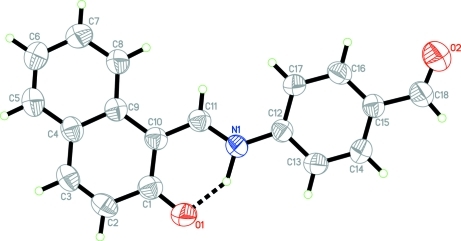
The asymmetric unit of the title compound, showing 50% probability displacement ellipsoids and the atom-numbering scheme. Intramolecular interaction is shown as dashed lines

**Fig. 2.**
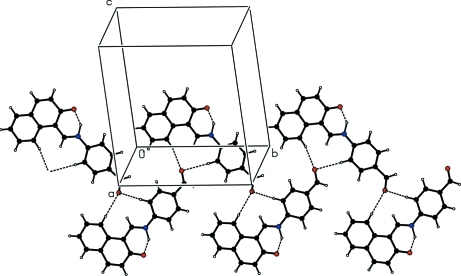
A molecular ribbon generated by C—H···O hydrogen bonds.

## supplementary crystallographic information

### Comment

Condensation reactions between carbonyl compounds and primary amines have provided one of the most important and widely studied classes of chelating ligand. The ligands, usually obtained via Schiff base condensation reactions, show variations in flexibility and electronic properties (Alsalim *et al.*, 2010). Schiff bases are a class of important compounds  in the medicinal and pharmaceutical fields. They exhibit biological properties, including antibacterial, antifungal (Wadher *et al.*, 2009; Abou-Melha & Faruk, 2008), anticancer, herbicidal (Wadher *et al.*, 2009), anti-inflammatory, analgesic and kinase inhibitory activities (Sondhi *et al.*, 2006).

The asymmetric unit of the title compound is shown in Fig. 1. The molecule is a zwitterion in the crystal, with the naphthol hydroxy group deprotonated and the imine N atom protonated. It adopts an *E* configuration about the central C11═N1 double bond [1.329 (3) Å] with the torsion angle C10-C11-N1-C12 = -179.4 (3)°. The dihedral angle between the naphthyl (C1–C10) ring system and the benzene (C12–C17) ring is 1.73 (11)°.

In the crystal structure (Fig. 2), an intramolecular N1—H1N1···O1 hydrogen bonding generates an *S*(6) ring motif (Bernstein *et al.*, 1995). Furthermore, the adjacent molecules are connected by intermolecular C8—H7···O2 and C17—H17A···O2 (Table 1) hydrogen bonds forming a supramolecular ribbon along the *b*-axis.

### Experimental

2-Hydroxy-1-naphthaldehyde (0.172 g, 1 mmol) was added to the solution of 4-aminobenzaldehyde (0.121 g, 1mmol) in ethanol (30 ml) following which the mixture was refluxed with stirring for 1 h. The resultant orange needle-shaped single crystals suitable for X-ray structure determination which formed was then filtered and washed with ethanol

### Refinement

Atom H1N1 was located from a difference Fourier map and refined freely [N—H = 1.00 (3) Å].  The remaining H atoms were positioned geometrically [C—H = 0.93 Å] and were refined using a riding model, with *U*_iso_(H) = 1.2 *U*_eq_(C).

### Crystal data

**Table:**

C_18_H_13_NO_2_	*F*(000) = 576
*M**_r_* = 275.29	*D*_x_ = 1.348 Mg m^−^^3^
Monoclinic, *P*2_1_/*c*	Mo *K*α radiation, λ = 0.71073 Å
Hall symbol: -P 2ybc	Cell parameters from 2561 reflections
*a* = 7.3685 (8) Å	θ = 3.2–30.0°
*b* = 12.7437 (13) Å	µ = 0.09 mm^−^^1^
*c* = 14.4586 (15) Å	*T* = 296 K
β = 91.979 (7)°	Needle, orange
*V* = 1356.9 (2) Å^3^	0.86 × 0.08 × 0.07 mm
*Z* = 4	

### Data collection

**Table:**

Bruker SMART APEXII CCD area-detector diffractometer	2394 independent reflections
Radiation source: fine-focus sealed tube	1416 reflections with *I* > 2σ(*I*)
graphite	*R*_int_ = 0.054
φ and ω scans	θ_max_ = 25.0°, θ_min_ = 2.1°
Absorption correction: multi-scan (*SADABS*; Bruker, 2009)	*h* = −8→8
*T*_min_ = 0.928, *T*_max_ = 0.994	*k* = −15→14
11574 measured reflections	*l* = −16→17

### Refinement

**Table:**

Refinement on *F*^2^	Primary atom site location: structure-invariant direct methods
Least-squares matrix: full	Secondary atom site location: difference Fourier map
*R*[*F*^2^ > 2σ(*F*^2^)] = 0.064	Hydrogen site location: inferred from neighbouring sites
*wR*(*F*^2^) = 0.171	H atoms treated by a mixture of independent and constrained refinement
*S* = 1.04	*w* = 1/[σ^2^(*F*_o_^2^) + (0.0799*P*)^2^ + 0.244*P*] where *P* = (*F*_o_^2^ + 2*F*_c_^2^)/3
2394 reflections	(Δ/σ)_max_ = 0.001
194 parameters	Δρ_max_ = 0.13 e Å^−^^3^
0 restraints	Δρ_min_ = −0.23 e Å^−^^3^

### Special details

**Table:**

Geometry. All esds (except the esd in the dihedral angle between two l.s. planes) are estimated using the full covariance matrix. The cell esds are taken into account individually in the estimation of esds in distances, angles and torsion angles; correlations between esds in cell parameters are only used when they are defined by crystal symmetry. An approximate (isotropic) treatment of cell esds is used for estimating esds involving l.s. planes.
Refinement. Refinement of F^2^ against ALL reflections. The weighted R-factor wR and goodness of fit S are based on F^2^, conventional R-factors R are based on F, with F set to zero for negative F^2^. The threshold expression of F^2^ > 2sigma(F^2^) is used only for calculating R-factors(gt) etc. and is not relevant to the choice of reflections for refinement. R-factors based on F^2^ are statistically about twice as large as those based on F, and R- factors based on ALL data will be even larger.

### Fractional atomic coordinates and isotropic or equivalent isotropic displacement parameters (Å^2^)

**Table:**

	*x*	*y*	*z*	*U*_iso_*/*U*_eq_	
O1	0.6211 (3)	−0.00216 (15)	0.67024 (13)	0.0694 (7)	
O2	0.9842 (4)	−0.31405 (18)	0.16917 (18)	0.0913 (8)	
N1	0.7418 (3)	−0.02531 (16)	0.50829 (17)	0.0496 (6)	
C1	0.6323 (4)	0.0967 (2)	0.65940 (19)	0.0527 (7)	
C2	0.5864 (4)	0.1658 (2)	0.73351 (19)	0.0578 (8)	
H2	0.5496	0.1373	0.7890	0.069*	
C3	0.5952 (4)	0.2702 (2)	0.72465 (19)	0.0563 (8)	
H3	0.5634	0.3119	0.7743	0.068*	
C4	0.6515 (4)	0.3201 (2)	0.64209 (19)	0.0503 (7)	
C5	0.6622 (4)	0.4289 (2)	0.6367 (2)	0.0673 (9)	
H4	0.6302	0.4692	0.6872	0.081*	
C6	0.7185 (5)	0.4774 (2)	0.5590 (2)	0.0804 (11)	
H5	0.7255	0.5502	0.5564	0.096*	
C7	0.7654 (5)	0.4169 (2)	0.4837 (2)	0.0752 (10)	
H6	0.8034	0.4496	0.4302	0.090*	
C8	0.7564 (4)	0.3100 (2)	0.4871 (2)	0.0587 (8)	
H7	0.7884	0.2712	0.4356	0.070*	
C9	0.7000 (3)	0.25724 (19)	0.56633 (17)	0.0450 (7)	
C10	0.6907 (3)	0.14332 (19)	0.57427 (17)	0.0448 (7)	
C11	0.7430 (3)	0.07881 (19)	0.50285 (18)	0.0463 (7)	
H11A	0.7807	0.1098	0.4485	0.056*	
C12	0.7929 (3)	−0.09668 (18)	0.44023 (18)	0.0440 (7)	
C13	0.7754 (4)	−0.20274 (19)	0.46048 (19)	0.0524 (7)	
H13A	0.7329	−0.2236	0.5175	0.063*	
C14	0.8209 (4)	−0.2768 (2)	0.39614 (19)	0.0537 (7)	
H14A	0.8092	−0.3476	0.4104	0.064*	
C15	0.8834 (3)	−0.24824 (19)	0.31103 (18)	0.0455 (7)	
C16	0.9013 (4)	−0.1417 (2)	0.29133 (19)	0.0534 (7)	
H16A	0.9443	−0.1212	0.2344	0.064*	
C17	0.8566 (4)	−0.0671 (2)	0.35455 (18)	0.0513 (7)	
H17A	0.8688	0.0037	0.3403	0.062*	
C18	0.9279 (4)	−0.3289 (2)	0.2450 (2)	0.0625 (8)	
H18A	0.9115	−0.3982	0.2629	0.075*	
H1N1	0.698 (4)	−0.048 (2)	0.570 (2)	0.086 (10)*	

### Atomic displacement parameters (Å^2^)

**Table:**

	*U*^11^	*U*^22^	*U*^33^	*U*^12^	*U*^13^	*U*^23^
O1	0.0902 (17)	0.0565 (13)	0.0634 (14)	0.0048 (11)	0.0285 (12)	0.0080 (10)
O2	0.116 (2)	0.0838 (16)	0.0757 (17)	−0.0014 (13)	0.0242 (15)	−0.0122 (12)
N1	0.0528 (15)	0.0482 (14)	0.0486 (15)	0.0021 (10)	0.0130 (12)	−0.0011 (11)
C1	0.0472 (18)	0.0561 (18)	0.0552 (18)	0.0041 (13)	0.0085 (14)	0.0025 (14)
C2	0.0542 (19)	0.074 (2)	0.0461 (17)	−0.0002 (15)	0.0125 (14)	−0.0011 (14)
C3	0.053 (2)	0.065 (2)	0.0511 (18)	0.0029 (14)	0.0072 (15)	−0.0126 (14)
C4	0.0432 (17)	0.0557 (17)	0.0520 (17)	0.0027 (12)	0.0037 (14)	−0.0088 (13)
C5	0.080 (2)	0.0574 (19)	0.065 (2)	0.0052 (15)	0.0082 (18)	−0.0145 (15)
C6	0.115 (3)	0.0487 (18)	0.078 (2)	0.0010 (17)	0.016 (2)	−0.0039 (17)
C7	0.104 (3)	0.055 (2)	0.068 (2)	0.0024 (17)	0.0191 (19)	0.0091 (16)
C8	0.072 (2)	0.0506 (18)	0.0543 (18)	0.0065 (14)	0.0099 (16)	−0.0014 (13)
C9	0.0373 (16)	0.0488 (15)	0.0487 (16)	0.0020 (11)	0.0018 (13)	−0.0020 (12)
C10	0.0379 (16)	0.0494 (16)	0.0475 (16)	0.0032 (11)	0.0058 (13)	−0.0001 (12)
C11	0.0440 (16)	0.0482 (16)	0.0470 (16)	0.0035 (12)	0.0053 (13)	0.0025 (12)
C12	0.0374 (16)	0.0447 (15)	0.0500 (17)	0.0019 (11)	0.0053 (13)	−0.0008 (12)
C13	0.0563 (19)	0.0534 (17)	0.0479 (16)	0.0013 (13)	0.0088 (14)	0.0058 (13)
C14	0.0603 (19)	0.0427 (15)	0.0581 (18)	0.0008 (12)	0.0048 (15)	−0.0005 (13)
C15	0.0385 (16)	0.0478 (15)	0.0501 (17)	0.0002 (12)	0.0022 (13)	−0.0032 (12)
C16	0.0525 (19)	0.0583 (18)	0.0500 (17)	−0.0030 (13)	0.0124 (14)	−0.0004 (13)
C17	0.0570 (18)	0.0440 (15)	0.0539 (17)	−0.0007 (12)	0.0152 (14)	0.0032 (12)
C18	0.071 (2)	0.0581 (18)	0.059 (2)	−0.0023 (15)	0.0134 (17)	−0.0067 (14)

### Geometric parameters (Å, °)

**Table:**

O1—C1	1.272 (3)	C7—H6	0.9300
O2—C18	1.201 (3)	C8—C9	1.404 (4)
N1—C11	1.329 (3)	C8—H7	0.9300
N1—C12	1.401 (3)	C9—C10	1.458 (3)
N1—H1N1	1.00 (3)	C10—C11	1.385 (3)
C1—C2	1.437 (4)	C11—H11A	0.9300
C1—C10	1.446 (3)	C12—C13	1.390 (3)
C2—C3	1.338 (4)	C12—C17	1.392 (3)
C2—H2	0.9300	C13—C14	1.374 (4)
C3—C4	1.427 (4)	C13—H13A	0.9300
C3—H3	0.9300	C14—C15	1.378 (4)
C4—C5	1.390 (4)	C14—H14A	0.9300
C4—C9	1.413 (3)	C15—C16	1.394 (3)
C5—C6	1.359 (4)	C15—C18	1.448 (4)
C5—H4	0.9300	C16—C17	1.367 (3)
C6—C7	1.389 (4)	C16—H16A	0.9300
C6—H5	0.9300	C17—H17A	0.9300
C7—C8	1.364 (4)	C18—H18A	0.9300
			
C11—N1—C12	127.2 (2)	C4—C9—C10	119.3 (2)
C11—N1—H1N1	110.3 (17)	C11—C10—C1	119.3 (2)
C12—N1—H1N1	122.6 (17)	C11—C10—C9	121.2 (2)
O1—C1—C2	119.8 (2)	C1—C10—C9	119.5 (2)
O1—C1—C10	122.3 (2)	N1—C11—C10	123.1 (2)
C2—C1—C10	117.9 (2)	N1—C11—H11A	118.5
C3—C2—C1	121.7 (3)	C10—C11—H11A	118.5
C3—C2—H2	119.2	C13—C12—C17	119.2 (2)
C1—C2—H2	119.2	C13—C12—N1	117.0 (2)
C2—C3—C4	122.7 (3)	C17—C12—N1	123.8 (2)
C2—C3—H3	118.7	C14—C13—C12	119.9 (2)
C4—C3—H3	118.7	C14—C13—H13A	120.1
C5—C4—C9	120.3 (3)	C12—C13—H13A	120.1
C5—C4—C3	120.7 (3)	C13—C14—C15	121.4 (2)
C9—C4—C3	119.0 (2)	C13—C14—H14A	119.3
C6—C5—C4	121.3 (3)	C15—C14—H14A	119.3
C6—C5—H4	119.3	C14—C15—C16	118.4 (2)
C4—C5—H4	119.3	C14—C15—C18	119.5 (2)
C5—C6—C7	119.1 (3)	C16—C15—C18	122.1 (3)
C5—C6—H5	120.5	C17—C16—C15	120.9 (2)
C7—C6—H5	120.5	C17—C16—H16A	119.5
C8—C7—C6	120.9 (3)	C15—C16—H16A	119.5
C8—C7—H6	119.6	C16—C17—C12	120.2 (2)
C6—C7—H6	119.6	C16—C17—H17A	119.9
C7—C8—C9	121.6 (3)	C12—C17—H17A	119.9
C7—C8—H7	119.2	O2—C18—C15	125.7 (3)
C9—C8—H7	119.2	O2—C18—H18A	117.2
C8—C9—C4	116.8 (2)	C15—C18—H18A	117.2
C8—C9—C10	123.9 (2)		
			
O1—C1—C2—C3	−179.6 (3)	C4—C9—C10—C11	−177.8 (2)
C10—C1—C2—C3	0.6 (4)	C8—C9—C10—C1	179.4 (3)
C1—C2—C3—C4	−0.4 (4)	C4—C9—C10—C1	−0.1 (3)
C2—C3—C4—C5	−178.7 (3)	C12—N1—C11—C10	−179.6 (2)
C2—C3—C4—C9	0.0 (4)	C1—C10—C11—N1	0.2 (4)
C9—C4—C5—C6	0.2 (5)	C9—C10—C11—N1	177.9 (2)
C3—C4—C5—C6	178.9 (3)	C11—N1—C12—C13	−178.2 (2)
C4—C5—C6—C7	0.3 (5)	C11—N1—C12—C17	0.8 (4)
C5—C6—C7—C8	−0.3 (5)	C17—C12—C13—C14	0.0 (4)
C6—C7—C8—C9	−0.1 (5)	N1—C12—C13—C14	179.1 (2)
C7—C8—C9—C4	0.6 (4)	C12—C13—C14—C15	−0.3 (4)
C7—C8—C9—C10	−179.0 (3)	C13—C14—C15—C16	0.5 (4)
C5—C4—C9—C8	−0.6 (4)	C13—C14—C15—C18	−179.4 (3)
C3—C4—C9—C8	−179.3 (2)	C14—C15—C16—C17	−0.5 (4)
C5—C4—C9—C10	178.9 (2)	C18—C15—C16—C17	179.3 (3)
C3—C4—C9—C10	0.2 (4)	C15—C16—C17—C12	0.3 (4)
O1—C1—C10—C11	−2.4 (4)	C13—C12—C17—C16	0.0 (4)
C2—C1—C10—C11	177.4 (2)	N1—C12—C17—C16	−179.1 (2)
O1—C1—C10—C9	179.8 (2)	C14—C15—C18—O2	−179.6 (3)
C2—C1—C10—C9	−0.3 (4)	C16—C15—C18—O2	0.5 (5)
C8—C9—C10—C11	1.7 (4)		

### Hydrogen-bond geometry (Å, °)

**Table:**

*D*—H···*A*	*D*—H	H···*A*	*D*···*A*	*D*—H···*A*
N1—H1N1···O1	1.00 (3)	1.68 (3)	2.551 (3)	143 (2)
C8—H7···O2^i^	0.93	2.54	3.399 (4)	153
C17—H17A···O2^i^	0.93	2.57	3.453 (4)	159

Symmetry codes: (i) −*x*+2, *y*+1/2, −*z*+1/2.
